# Conserved *Hepatospora eriocheir* Sequences in Chinese Mitten Crabs from Poland, Germany and Belgium: A Comparative Molecular Study

**DOI:** 10.3390/ijms27219646

**Published:** 2026-10-28

**Authors:** Magdalena Stachnik, Monika Normant-Saremba, Christine Ewers, Heleen Keirsebelik, Jonas Schoelynck

**Affiliations:** 1 Department of Parasitology and Invasive Diseases, Bee Diseases and Aquatic Animal Diseases, National Veterinary Research Institute, Al. Partyzantów 57, 24-100 Puławy, Poland; 2 Department of Marine Ecology, Faculty of Oceanography and Geography, University of Gdańsk, Al. M. Piłsudskiego 46, 81-378 Gdynia, Poland; 3 Center for Molecular Biodiversity, Leibniz Institute for the Analysis of Biodiversity Change, Bundesstrasse 43, 20146 Hamburg, Germany; c.ewers@leibniz-lib.de; 4 ECOSPHERE Research Group, Department of Biology, University of Antwerp, Universiteitsplein 1C, 2610 Antwerp, Belgium; heleen.keirsebelik@uantwerpen.be (H.K.); jonas.schoelynck@uantwerpen.be (J.S.)

**Keywords:** *Hepatospora eriocheir*, *Eriocheir sinensis*, microsporidia, ArgRS, SSU rRNA, Chinese mitten crab, North Sea, Baltic Sea, host mitochondrial COI

## Abstract

*Hepatospora eriocheir* is an obligate intracellular microsporidian associated with the Chinese mitten crab, *Eriocheir sinensis*, an important aquaculture species in Asia and an invasive species in Europe. We investigated parasite sequence variation in crabs from three European regions: the Vistula Lagoon in the Baltic Sea (Poland) and the Elbe and Scheldt catchments draining into the North Sea (Germany and Belgium). Among 57 crabs examined, 26 infections were confirmed by small subunit ribosomal RNA (SSU rRNA) sequencing, including 17 newly identified infections among 42 specimens collected in 2022. The parasite groups comprised 13 Polish, eight German and five Belgian infections. Their SSU rRNA sequences were identical across a 794-position shared core, and all 26 newly generated arginyl-tRNA synthetase (ArgRS) sequences were identical across 624 nucleotides and matched the British reference KU695717. Both parasite gene fragments were identical across four host mitochondrial cytochrome c oxidase subunit I (COI) haplotypes; the *Eriocheir japonica*-associated haplotype was detected only in Belgian crabs in this sample. The study provides the first specimen-linked, sequence-confirmed molecular characterisation of infections in the sampled German and Belgian crab populations, together with new Polish infections from 2022 and a comparative two-locus reference dataset. The repeated Baltic detection, combined with shared Polish–German host COI haplotypes and existing evidence of North Sea–Baltic connectivity, is consistent with the arrival of infected migrating crabs, although the parasite’s precise source remains unresolved.

## 1. Introduction

The geographical expansion of invasive hosts can alter the distribution of their associated parasites and host–parasite interactions in recipient ecosystems, with implications for aquatic animal health and biological invasions [1,2]. Sequence-based detection is particularly valuable where earlier records are sparse or diagnostics differ. The Chinese mitten crab, *Eriocheir sinensis*, is an economically important aquaculture species in East Asia [3] and an invasive alien species of Union concern in Europe [4]. Its catadromous life cycle involves freshwater growth followed by downstream reproductive migration towards saline waters [5,6,7], connecting habitats in which host-associated pathogens may be transported. Nevertheless, the geographical distribution and molecular diversity of its parasites remain incompletely characterised.

The obligate intracellular microsporidian *Hepatospora eriocheir* infects hepatopancreatic epithelial cells of *E. sinensis* and is associated with substantial disease-related concerns in aquaculture [8,9,10,11,12,13,14]. Outbreaks of hepatopancreatic necrosis disease in Chinese crab farms, where H. eriocheir-like microsporidia were detected, were accompanied by reported mortality of 40–50% [8]. However, the contribution of the parasite to all observed deaths has not been established. Originally described from Chinese mitten crabs in Asia, *H. eriocheir* was subsequently characterised in invasive crabs from the Thames estuary, United Kingdom [8,15]. Later European physiological investigations have associated infection with reduced aerobic performance and altered energy reserves [16], although consequences for host population dynamics remain unresolved. Histopathology may reveal epithelial degeneration, tubular disruption, spore accumulation and necrosis [15]. However, gross lesions are not specific, and mild infections may lack conspicuous tissue changes. Parasite-gene sequencing can confirm identity but not disease severity or viability.

Following the initial European description [15], Bateman et al. [17] analysed SSU rRNA and six protein-coding loci in *Hepatospora* associated with *E. sinensis*, *Cancer pagurus* and *Pinnotheres pisum*. Related draft genome assemblies were subsequently reported by Wiredu Boakye et al. [18]. Among the six coding genes, arginyl-tRNA synthetase (ArgRS) exhibited the highest proportion of variable nucleotide positions in comparisons between the host-associated parasite lineages [17]. This provided a defined rationale for pairing ArgRS with small subunit ribosomal RNA (SSU rRNA) in the present comparison, although inter-host differences do not establish ArgRS as a geographic population marker within *H. eriocheir*. The only comparable published *E. sinensis*-associated ArgRS reference was British sequence KU695717 [17], rather than a replicated European series. Furthermore, suitable isolate-level SSU rRNA and ArgRS sequences from Chinese-origin infections could not be verified among the public records consulted, restricting comparisons between native- and introduced-range material. Closely related *Hepatospora* from other crab hosts also leave unresolved questions concerning host range and historical host switching.

Our previous study molecularly confirmed *H. eriocheir* in Chinese mitten crabs collected from the Vistula Lagoon in 2020, providing the first published record of this parasite in the Baltic Sea [19]. This finding prompted a search for infected hosts in North Sea catchments potentially connected to Baltic arrivals. Low salinity constrains successful larval development in much of the south-eastern Baltic [5]. A self-sustaining population of *E. sinensis* has not been demonstrated at the Vistula Lagoon locality, although mating or embryonic development has been reported elsewhere in the southern Baltic [20,21]. Ecological and genetic evidence points to connectivity between Baltic crabs and North Sea drainage populations, particularly in the Elbe system [5,7,22]. Accordingly, samples were collected in 2022 from the Elbe in Germany, the Kleine Nete within the Scheldt catchment in Belgium and the Vistula Lagoon in Poland. This design examines parasite occurrence without tracing individual crab origins.

We combined the previously investigated Polish cohort from 2020 with new material collected in 2022, partly because adult crabs are obtained only irregularly from the Polish Baltic coast. Our study had three objectives. First, we sought to obtain sequence-confirmed evidence of *H. eriocheir* infection in the German and Belgian North Sea drainage populations. Second, we investigated whether infected crabs were again present in the Vistula Lagoon in 2022, consistent with continued parasite occurrence in an area without demonstrated local host recruitment. Third, we compared parasite SSU rRNA and ArgRS sequence types across geographic groups and host mitochondrial cytochrome c oxidase subunit I (COI) haplotypes, including the Eriocheir japonica-associated lineage reported from Europe [22,23]. This comparative baseline cannot alone establish historical introduction routes.

## 2. Results

### 2.1. Detection of Hepatospora eriocheir

Among 42 *E. sinensis* collected in 2022, SSU rRNA sequencing confirmed 17 infections: four of nine Polish, eight of 18 German and five of 15 Belgian crabs (Table 1). We additionally included 15 Polish crabs collected in October 2020, nine of which were previously reported positive [19]. The combined dataset therefore comprised 26 sequence-confirmed infections among 57 examined crabs. New ArgRS sequences were generated for all 26 infected individuals, including the previously published Polish cohort. The present study provides paired parasite SSU rRNA and ArgRS data from the sampled German and Belgian populations for the first time; it does not claim the first report of parasite occurrence in either country.

**Figure 1:**
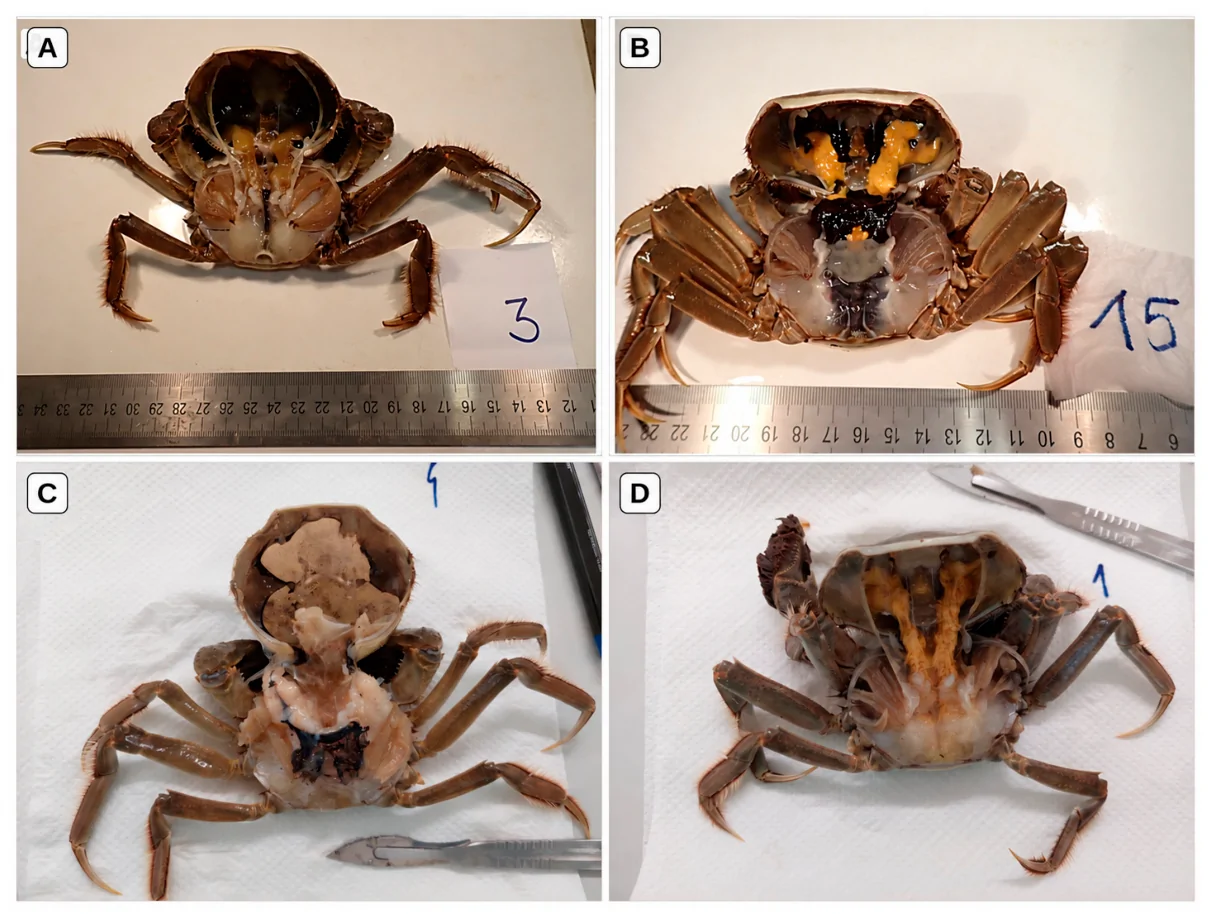
Representative macroscopic appearance of the hepatopancreas in *Eriocheir sinensis* from Belgium and Germany sampled in 2022. (**A**) *Hepatospora eriocheir*-positive Belgian specimen BE3; (**B**) *H. eriocheir*-negative Belgian specimen BE15; (**C**) *H. eriocheir*-positive German specimen DE4; (**D**) *H. eriocheir*-negative German specimen DE1. Infection status was determined by PCR followed by SSU rRNA amplicon sequencing, independently of gross appearance.

Gross hepatopancreatic morphology varied from compact yellow or yellow-orange tissue to paler, discoloured and less compact tissue with partial loss of the normal lobular appearance. Comparable gross variation was observed in the 2022 German and Belgian material and in the Polish series; representative Belgian and German specimens are shown in Figure 1. However, the presence or absence of visible hepatopancreatic changes did not consistently correspond to parasite infection status. Gross examination alone was therefore insufficient for reliable identification of infected crabs, and molecular confirmation by PCR was required. Molecular status was assigned independently by sequence-confirmed detection of *H. eriocheir*. Gross appearance was not used as a diagnostic criterion or as a quantitative measure of disease severity. Among the illustrated individuals, SSU rRNA-positive Belgian crab BE3 and German crab DE4 had a relatively pale, whitish-beige hepatopancreas, whereas the negative specimens BE15 and DE1 showed more yellow-orange pigmentation (Figure 1A–D). The organ may become pale and friable in advanced infections, but tissue colour and macroscopic appearance are non-specific and early or low-intensity infections may not produce conspicuous lesions [15,19]. These photographs are descriptive and cannot establish infection status, parasite burden or the severity of histological injury.

### 2.2. Host COI Haplotypes and Geographic Context

For comparative analyses, all COI sequences were trimmed to a common homologous 624-bp fragment shared by all 57 crabs. Based on exact sequence identity across this standardised fragment, each sequence was assigned to one of four published mitochondrial haplotypes: ES_HP1, ES_HP2, ES_HP4 or the *E. japonica*-associated lineage EJJ_HP14. ES_HP1 was the most frequent haplotype (23/57), followed by ES_HP2 (20/57), EJJ_HP14 (9/57) and ES_HP4 (5/57). The Polish and German samples contained ES_HP1, ES_HP2 and ES_HP4 and showed closely comparable haplotype composition. In Belgium, EJJ_HP14 occurred in 9/15 crabs (60.0%). Across the 624-bp alignment, ES_HP1, ES_HP2 and ES_HP4 differed pairwise by two substitutions, whereas EJJ_HP14 differed by 27 substitutions from each of these *E. sinensis*-associated haplotypes. This divergent *E. japonica*-associated mitochondrial lineage is consistent with introgression, as supported by published morphological and nuclear-genomic evidence, and does not demonstrate a separate nuclear host species [22,23].

Haplotype diversity (Hd) was 0.649 in Poland, 0.569 in Germany and 0.562 in Belgium, whereas nucleotide diversity (π) was 0.00208, 0.00182 and 0.02241, respectively (Table 2). The higher Belgian nucleotide diversity reflects the occurrence of the divergent EJJ_HP14 mitochondrial lineage. Given the small, unevenly distributed samples and the pooling of two sampling years in Poland, these descriptive indices were not used to infer host population structure or migration routes.

### 2.3. Parasite SSU rRNA and ArgRS Sequence Comparison

**Figure 2:**
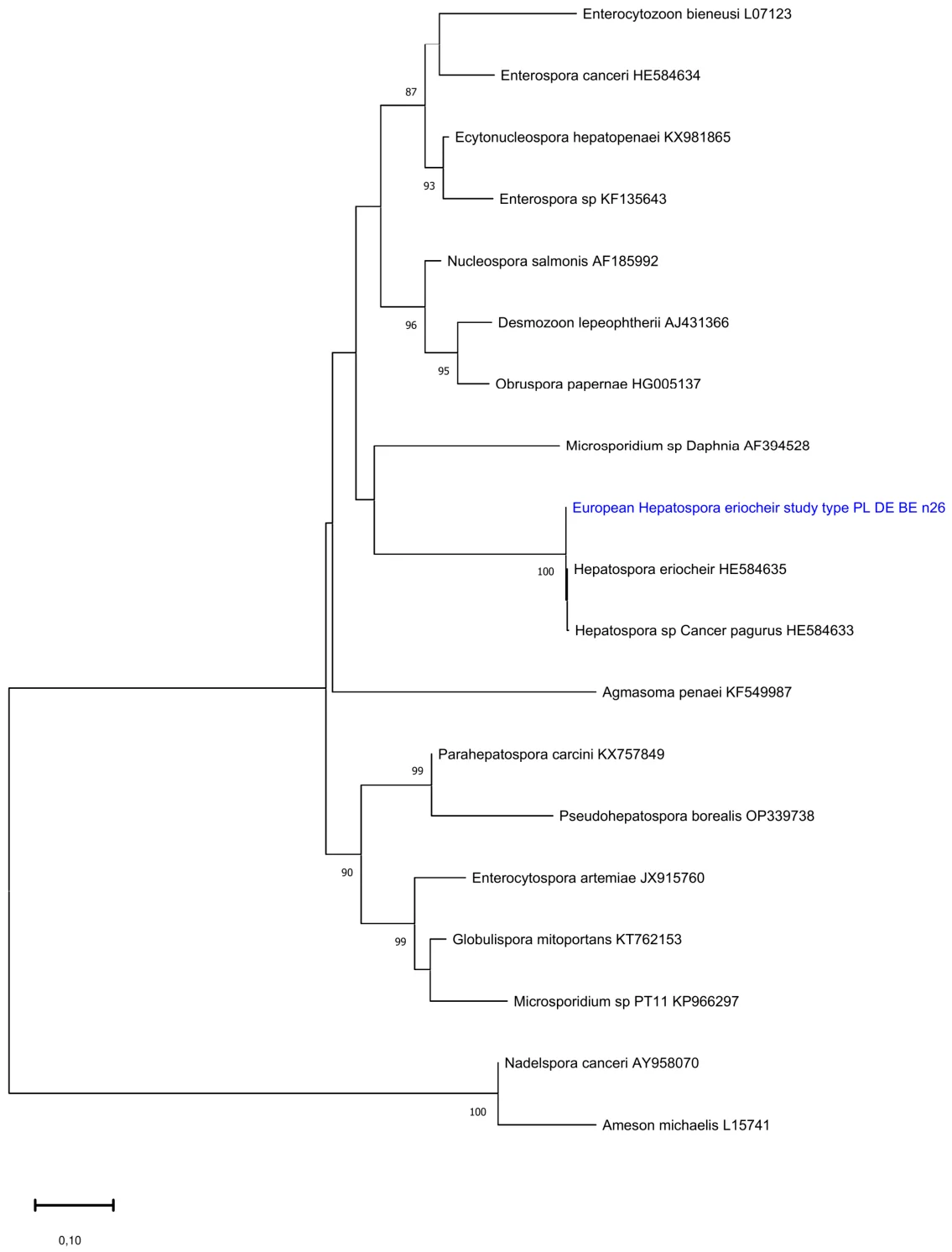
Maximum-likelihood phylogenetic placement of a representative *Hepatospora eriocheir* small subunit ribosomal RNA (SSU rRNA) sequence shared by 26 infected crabs from Poland, Germany and Belgium, relative to 18 published microsporidian reference sequences. The TN93+G model (Tamura–Nei 1993 with gamma-distributed rate variation) was applied to 499 aligned nucleotide positions with 1000 bootstrap replicates; only support values of at least 80% are shown. The tree was midpoint rooted. The scale bar indicates 0.10 substitutions per site. Reference accessions are listed in Supplementary Table S2.

The 26 parasite SSU rRNA sequences were identical throughout a shared alignment of 794 unambiguous positions, with no differences within or between the Polish (*n* = 13), German (*n* = 8) and Belgian (*n* = 5) infected groups. The study sequence differed by one nucleotide from the British *H. eriocheir* reference HE584635 across 789 comparable positions (99.87% identity) and matched the shorter *Hepatospora* sequence HE584633 from *C. pagurus* across 504 positions. Maximum-likelihood analysis of a 499-position reference alignment placed the representative study sequence among published *Hepatospora* references (Figure 2). Because the study sequences were identical, this analysis could not distinguish infections by sampling locality.

**Figure 3:**
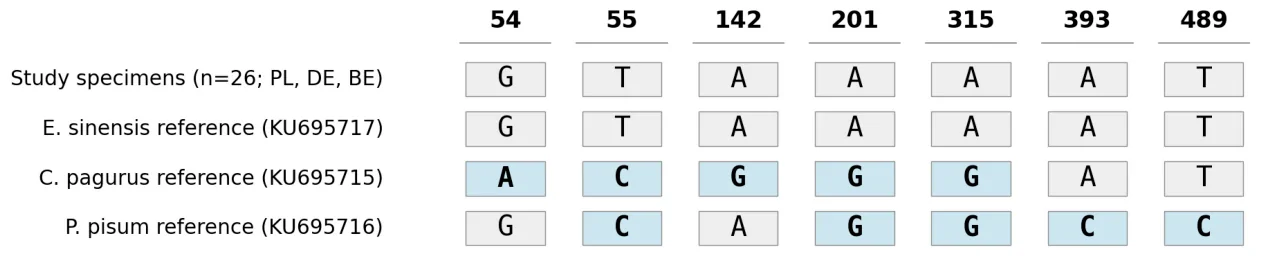
Comparison of seven variable sites within a 624-nucleotide fragment of arginyl-tRNA synthetase (ArgRS). Reference sequences were KU695717 (*Hepatospora* from *Eriocheir sinensis*), KU695715 (*Cancer pagurus*) and KU695716 (*Pinnotheres pisum*). All 26 sequences newly generated from the sampled infections in Poland (PL), Germany (DE) and Belgium (BE) were identical to KU695717 across the entire analysed fragment. Shaded positions represent substitutions among the published host-associated reference sequences, not variation among the 26 study samples. Grey cells indicate nucleotides identical to the *E. sinensis*-associated reference sequence, whereas blue cells highlight nucleotide substitutions relative to this reference. Numbers above the columns indicate nucleotide positions within the analysed fragment, and letters (A, C, G, T) represent the corresponding nucleotides.

ArgRS amplification and bidirectional sequencing yielded a 624-nt fragment for each of the 26 infections confirmed by SSU rRNA. Sequences from Poland (*n* = 13), Germany (*n* = 8) and Belgium (*n* = 5) were identical to one another and to the British *E. sinensis*-associated reference KU695717. The reference types KU695715 (*C. pagurus*) and KU695716 (*P. pisum*) each differed from KU695717 at five of 624 positions, whereas KU695715 and KU695716 differed at four positions. The seven nucleotide sites variable among these reference sequences are shown in Figure 3. No variation within the study ArgRS dataset was detected.

No sequence variation associated with geographic origin or host mitochondrial haplotype was detected within the analysed fragment. The identical ArgRS profile across all 26 study samples, including parasites detected in crabs carrying ES_HP1, ES_HP2, ES_HP4 and EJJ_HP14 mitochondrial haplotypes, provided independent sequence support for their assignment to *H. eriocheir*. The concordant SSU rRNA and ArgRS results strongly support molecular assignment of the microsporidian detected in the European Chinese mitten crab populations to *H. eriocheir*.

### 2.4. Individual-Level Host–Parasite Comparison

Infected crabs occurred in four host mitochondrial backgrounds represented in the study. Sequence-confirmed infection was detected in each observed host COI haplotype: ES_HP1 (11/23), ES_HP2 (10/20), ES_HP4 (1/5) and EJJ_HP14 (4/9; Table 3). A two-sided Fisher–Freeman–Halton exact test found no statistically significant association between host COI haplotype and infection status (*p* = 0.712); however, the small and unbalanced groups do not establish equal infection probabilities. In Belgium, parasites from infected EJJ_HP14 and *E. sinensis*-associated mitochondrial backgrounds had identical SSU rRNA and ArgRS sequences.

Individual crab identifiers linked parasite SSU rRNA and ArgRS with host COI (Supplementary Table S1). The lack of parasite variation at both loci precludes meaningful parasite-population structure, migration-direction or host–parasite cophylogenetic analyses.

## 3. Discussion

The principal novelty of this study is the first specimen-linked, sequence-based confirmation and paired SSU rRNA–ArgRS characterisation of *H. eriocheir* infections in *E. sinensis* collected from two North Sea drainage systems: the German Elbe and Belgian Scheldt catchments. These results complement new molecular records from the Vistula Lagoon in 2022 and extend the Baltic observations from the 2020 collection [19]. Among 57 examined crabs, 26 infections were sequence-confirmed: 13 from Poland, eight from Germany and five from Belgium. Seventeen infections were newly detected in the 2022 samples; the remaining nine SSU rRNA-positive Polish individuals had been reported previously. All 26 ArgRS sequences were generated for the present study and linked to parasite SSU rRNA and host COI records for the same crabs. Prior accessible *E. sinensis*-associated ArgRS sequence comparisons relied on the British reference KU695717 [17]. The advance is therefore not limited to additional ArgRS sequences: it includes previously unavailable sequence-confirmed records from the sampled German and Belgian populations, newly documented Polish infections from 2022, and traceable paired parasite-marker and host-COI data. These results establish a comparative reference without implying that the parasite’s introduction history has been reconstructed.

Observed sequence-confirmed detection proportions were 33.3% in Belgium, 44.4% in Germany, and 44.4% and 60.0% in Polish collections from 2022 and 2020, respectively. Kerr et al. [24] reported histological detection of *H. eriocheir* in 8 of 39 Chinese mitten crabs (20.5%) collected from the Thames in 2018, without generating new parasite sequence records. Histopathology and sequence-confirmed molecular detection address infection using different evidence, and low-intensity infections without conspicuous lesions may be missed by morphology-based assessments. Conversely, PCR detection establishes the presence of target parasite DNA in the examined material but does not quantify disease severity, viability or clinical impact. Therefore, differences in detection method, sampling season, effort and sample size preclude interpreting these proportions as regional prevalence differences. Kerr et al. [24] also identified *Hematodinium perezi* genotype I molecularly in the Thames population, illustrating the broader parasite assemblage of introduced mitten crabs, without demonstrating host switching by *H. eriocheir*.

The choice of ArgRS was grounded in the multilocus comparison by Bateman et al. [17]. Of the six protein-coding genes examined, ArgRS showed the highest proportion of variable nucleotide positions in each comparison among *Hepatospora associated* with *E. sinensis*, *C. pagurus* and *P. pisum*: 21/669 (3.14%), 23/1064 (2.16%) and 20/669 (2.99%), respectively. These comparisons involved different fragment lengths and host-associated parasite lineages and did not predict geographic variability within *H. eriocheir*. The two loci serve complementary purposes: SSU rRNA provides phylogenetic placement, while ArgRS allows an independent protein-coding comparison with published host-associated reference sequences. Neither marker resolved geographic differences in the specimens examined, so the analysis provides a molecular reference dataset rather than a parasite population-genetic reconstruction. Earlier British sequence and genome resources [17,18] supply important references, whereas our study contributes an individually linked geographic series from Poland, Germany and Belgium.

All 26 infections showed identical parasite sequences across the 794-position shared SSU rRNA core and the 624-nt ArgRS fragment. The ArgRS sequences also matched the British *E. sinensis*-associated reference KU695717; homologues associated with *C. pagurus* and *P. pisum* differed from that reference at five positions within the analysed fragment. The seven informative sites among these reference types are summarised in Figure 3. The present findings therefore document one observed sequence type at each examined locus across the three geographic samples, rather than proving genome-wide clonality or a single introduction. This distinction is especially important because conserved gene regions, low diversity in source populations, repeated arrival of the same sequence type and other demographic histories can produce the same pattern. Bidirectional Sanger consensus sequences also do not exclude low-frequency within-host variants.

Studies of other introduced crustaceans and their microsporidia show why the interpretation requires caution. Host and parasite introductions can exhibit different genetic patterns [1], and associations involving *Cucumispora dikerogammari* and microsporidia of invasive amphipods have prompted hypotheses involving co-transport and host shifts [2,25]. These are useful comparative examples, not evidence that the same process occurred in *H. eriocheir*. The absence of verified, directly comparable Chinese-origin SSU rRNA and ArgRS isolate records prevents a native-range versus introduced-range diversity test. Discriminating among these histories will require broader sampling across Asian and European host populations and markers with greater within-species resolution. Genes encoding polar-tube and spore-wall proteins may be considered alongside genome-wide variants, although their discriminatory power within *H. eriocheir* has yet to be established.

The geographic sequence comparison also addresses the ecological question that motivated our continuation of the 2020 Baltic study. *H. eriocheir* was detected again in Vistula Lagoon crabs collected in 2022, and the Polish and German hosts shared COI haplotypes, in agreement with earlier evidence of connectivity among the Elbe, Oder and Vistula systems [5,7,22]. Because a self-sustaining population of *E. sinensis* has not been demonstrated at the sampled Baltic locality [5,20,21], carriage of the parasite by immigrating infected crabs is a plausible working explanation for its repeated occurrence. The molecular confirmation of infections in the Elbe and Scheldt catchments establishes that infected hosts occur in these North Sea drainage systems. Nevertheless, neither shared host mtDNA nor identical parasite fragments identify Germany or Belgium as the direct source of the Baltic infections. They also cannot distinguish one introduction from recurrent movements or rule out local transmission after arrival.

The relationship between parasite sequence conservation and host mitochondrial variation offers a further reference point. Four host COI haplotypes were detected, including the *E. japonica*-associated EJJ_HP14 type in Belgian specimens. Earlier genomic investigations support mitochondrial introgression among European Eriocheir lineages [22,23], so this mitochondrial type should not be interpreted as evidence of a separate nuclear species. Infected crabs carrying EJJ_HP14 and E. sinensis-associated haplotypes nevertheless shared the same parasite SSU rRNA and ArgRS profiles. This observation establishes that the detected sequence type occurs across more than one host mitochondrial background in our sample, but small subgroup sizes preclude strong inference about susceptibility or host–parasite evolutionary associations.

The potential contribution of alternative crab hosts requires further investigation. Bateman et al. [17] detected closely related *Hepatospora* lineages in the European marine crabs *C. pagurus* and *P. pisum*, demonstrating that related parasites occur beyond *E. sinensis* while leaving their species boundaries and host ranges unresolved. Several native and introduced decapods occur in North Sea and Baltic habitats, including the invasive *Rhithropanopeus harrisii* [26]. No *H. eriocheir* infection has been confirmed in *R. harrisii* or other Baltic crab species in the available records, but systematic screening is lacking; this absence of reports cannot be interpreted as evidence of absence. The intracellular life cycle and documented association with *E. sinensis* are consistent with host-associated transport, although exclusive host specificity remains unproven.

The 2022 collections were obtained during autumn, a period broadly overlapping the downstream reproductive migration of adult mitten crabs, but capture methods and sampling effort differed among sites [6,27,28]. Limited and irregular availability of adult *E. sinensis* along the Polish Baltic coast constrained the sample size [20]. The proportions in Table 1 consequently describe the animals examined rather than unbiased regional prevalence, and the non-significant infection comparisons among host COI haplotypes do not establish similar infection risks. The hepatopancreatic changes illustrated in Figure 1 provide morphological context, not a measure of infection intensity or proof of clinical disease.

The molecular confirmation of *H. eriocheir* in German and Belgian crabs sampled in 2022 prompted subsequent collaborative investigations into the physiological correlates of infection. In a separate group of crabs collected in October 2023, Keirsebelik [16] reported associations between infection and reduced maximum metabolic rate, aerobic scope and haemolymph glucose. Reduced hepatopancreatic lipid reserves were also associated with infection in the German material, but not in the Belgian material. These subsequent findings complement the present molecular records but were obtained from different animals. No physiological measurements were made in the present study. Whether infection directly affects migratory performance, reproduction or population dynamics remains to be established.

## 4. Materials and Methods

### 4.1. Study Design and Sample Collection

**Figure 4:**
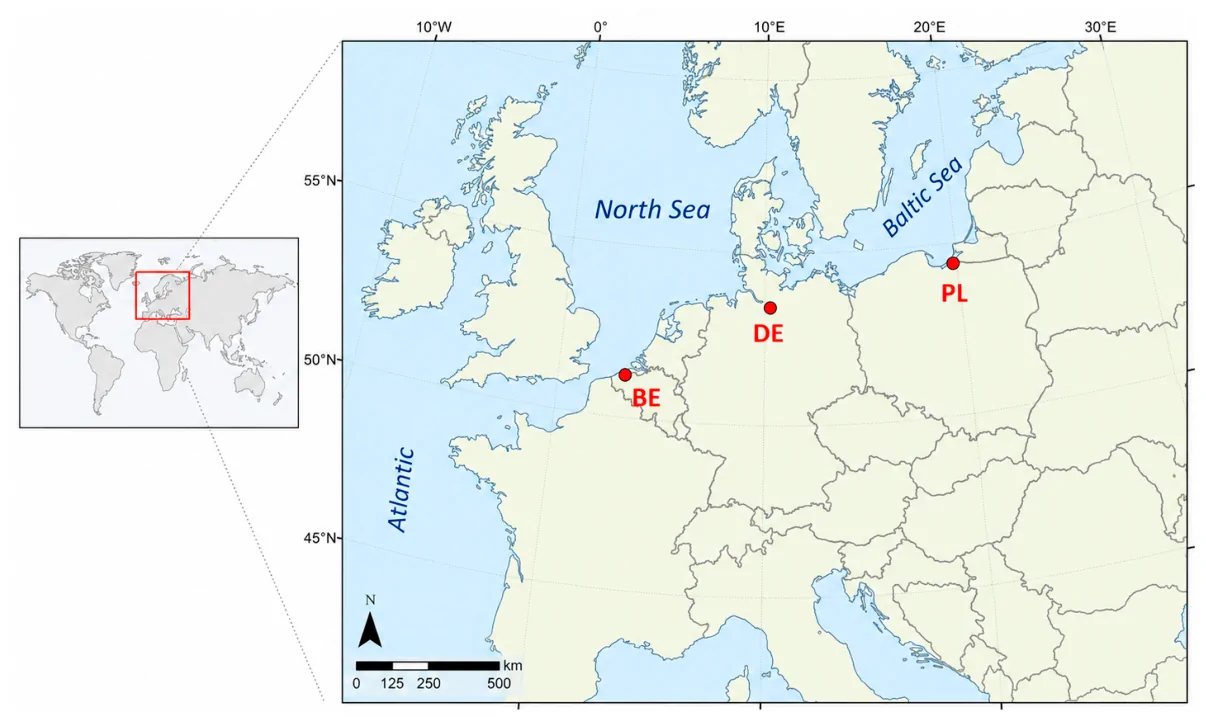
Sampling sites of the Chinese mitten crab *Eriocheir sinensis*: Vistula Lagoon near Frombork, Poland (PL; Baltic Sea, 2020 and 2022); Elbe River at Hohnstorf, Germany (DE; North Sea drainage, 2022); and Kleine Nete at Grobbendonk, Belgium (BE; Scheldt catchment draining to the North Sea, 2022).

The comparative dataset comprised 57 adult *E. sinensis* from three European regions. Fifteen crabs were collected in October 2020 from the south-eastern Vistula Lagoon, Poland, between Tolkmicko and Frombork. These specimens were obtained during commercial fishing with fyke nets and constitute the biological dataset reported previously in the first Baltic record of *H. eriocheir* [19]. They are included here only as a clearly identified reference component for expanded cross-country analyses and are not presented as new observations. In 2022, nine additional adult crabs were collected in November from the Frombork area of the Vistula Lagoon, Poland. In Germany, 18 crabs were collected in September from the Elbe River at Hohnstorf. In Belgium, 15 crabs were collected in November from the Kleine Nete River at Grobbendonk in the Scheldt basin. Polish and German crabs were obtained from local fishers using fyke nets, whereas Belgian crabs were captured using a dedicated Chinese mitten crab trap [27]. Carapace width was measured to the nearest 0.1 mm and used as the body-size metric. Mean carapace width was 65.7 mm in Poland in 2020, 66.8 mm in Poland in 2022, 58.3 mm in Germany in 2022 and 66.1 mm in Belgium in 2022. The sex composition was 8 males and 7 females in Poland in 2020, 5 males and 4 females in Poland in 2022, 10 males and 8 females in Germany in 2022 and 9 males and 6 females in Belgium in 2022. Sex and carapace width were recorded descriptively. Individual specimen identifiers and sequence accession links are supplied in Supplementary Table S1; sampling localities are shown in Figure 4. Capture methods were adapted to local hydromorphological conditions and available sampling infrastructure. Because year, locality and capture method were not fully balanced among regions, molecular detection frequencies were summarised by sampling group and between-group comparisons were treated as exploratory.

Following transport to the laboratory, crabs were rendered insensible by hypothermic chilling in ice water and then euthanised by immediate destruction of the nerve ganglia by spiking with a sharp instrument. The specimens were subsequently dissected under aseptic conditions. Tissue samples intended for molecular analyses were immediately preserved in 80% ethanol until DNA extraction, which was performed as described previously by Stachnik et al. [19]. During dissection, the hepatopancreas was also examined macroscopically for colour, tissue compactness or friability, and preservation of the normal lobular appearance. Representative photographs were retained, with selected Belgian and German examples shown in Figure 1. Macroscopic findings were recorded descriptively, whereas *H. eriocheir* infection status was determined independently by sequence-confirmed PCR detection. Accordingly, the terms infected and uninfected in Figure 1 refer exclusively to molecularly determined infection status. Gross hepatopancreatic appearance was not used as a diagnostic criterion or as a quantitative measure of disease severity.

### 4.2. Molecular Detection and Multilocus Characterisation of H. eriocheir

For the Polish 2020 specimens, DNA extraction, SSU rRNA screening, Sanger sequencing and host COI sequencing were completed and reported previously by Stachnik et al. [19]. Those SSU rRNA and COI records were retained for comparison, whereas new ArgRS sequences were generated from the same nine previously confirmed infections in the present study. For specimens collected in 2022, genomic DNA was extracted from hepatopancreatic tissue and walking-leg muscle using the QIAamp DNA Mini Kit (Qiagen, Hilden, Germany) according to the manufacturer’s instructions. Initial microsporidian screening of hepatopancreatic DNA used the MF1 (5′-CCGGAGAGGGAGCCTGAGA-3′) and MR1 (5′-GACGGGCGGTGTGTACAAA-3′) primers described by Tourtip et al. [29]. Because MF1/MR1 target conserved SSU rRNA regions, they were used as broad-range microsporidian screening primers rather than as a species-specific assay. PCR amplification used an annealing temperature of 55 °C [29]. Amplicons of the expected size were sequenced bidirectionally by Sanger sequencing at Genomed Joint-Stock Company (Warsaw, Poland). Forward and reverse chromatograms were inspected manually, assembled into consensus sequences and trimmed to remove low-quality terminal regions. Molecular positivity was assigned from high-quality SSU rRNA amplicon sequences attributable to Hepatospora, rather than from PCR product size alone. All 26 SSU rRNA-positive specimens were subsequently sequenced at ArgRS, providing an independent protein-coding reference comparison.

PCR amplifications newly performed for this study were carried out using Platinum Taq DNA Polymerase (Invitrogen, Thermo Fisher Scientific, Waltham, MA, USA) on a C1000 Touch thermal cycler (Bio-Rad Laboratories, Hercules, CA, USA). Each reaction had a final volume of 50 µL, comprising 45 µL of reaction mixture and 5 µL of template DNA. The reaction mixture followed the manufacturer’s standard formulation for a 50 µL reaction: 1× PCR buffer (without MgCl_2_), 1.5 mM MgCl_2_, 0.2 mM of each dNTP, 0.2 µM of each primer, 1 U of Platinum Taq DNA Polymerase and nuclease-free water to the required volume. The amplification began with initial denaturation at 94 °C for 2 min. This was followed by 35 cycles of denaturation at 94 °C for 30 s, annealing at 55 °C for 30 s for both SSU rRNA and ArgRS assays, and extension at 72 °C for 1 min per kb. The reactions were then held at 4 °C. A negative extraction control containing sterile water processed through the DNA extraction procedure and a no-template PCR control containing reaction mixture with sterile water instead of sample DNA were included to monitor potential contamination.

The SSU rRNA comparison included nine Polish 2020 sequences published previously [19] and 17 newly confirmed infections from specimens collected in 2022. Consensus sequences were aligned using MUSCLE [30]. Following terminal trimming and removal of one ambiguous alignment column, the within-study comparison contained 794 homologous unambiguous positions shared by all 26 specimens. This unambiguous core, not the taxonomic 80–coverage alignment, was used for within-study invariance. SSU rRNA consensus sequences were assembled from bidirectional Sanger reads; low-frequency within-host alleles could not be resolved.

For phylogenetic placement, one representative study SSU rRNA sequence was aligned to a panel of 18 published microsporidian references, including HE584635 (*H. eriocheir*) and HE584633 (*Hepatospora* sp. from *Cancer pagurus*) [17]. Removing sites with fewer than 80% unambiguous nucleotides left 499 positions. Maximum-likelihood analysis in MEGA 11 [31] used TN93+G with 1000 bootstrap replicates. Reference identities were calculated on pairwise unambiguous overlaps, and a midpoint root was used only for tree display (Figure 2; Supplementary Table S2).

The arginyl-tRNA synthetase gene (ArgRS) was sequenced for all 26 SSU rRNA-positive individuals, including nine crabs from the previously published Polish 2020 cohort. The F1 (5′-ATGGAAGAAGGTATAAATGAGG-3′) and R1 (5′-GGTCCCAGTGATTCACCTTT-3′) primers described by Bateman et al. [17] were used with an annealing temperature of 55 °C. Bidirectional Sanger reads were trimmed to a shared 624-nt coding fragment (208 codons), which was translated to verify the reading frame. The study sequences were compared with KU695717 (*E. sinensis*), KU695715 (*C. pagurus*) and KU695716 (*P. pisum*). Pairwise nucleotide substitutions were counted across the shared alignment, and seven reference-variable positions were displayed (Figure 3). ArgRS was selected on the basis of its comparatively high proportion of variable sites among six genes analysed across different crab-associated *Hepatospora* by Bateman et al. [17], without assuming corresponding variability among geographic groups of *H. eriocheir*. No population phylogeny was estimated from the 26 identical study sequences.

### 4.3. Host COI Amplification, Sequencing and Haplotype Assignment

For the crabs collected in 2022, mitochondrial cytochrome c oxidase subunit I (COI) was amplified from walking-leg muscle DNA with the universal primers LCO1490 and HCO2198 [32]. PCR was performed using Platinum Taq DNA Polymerase (Invitrogen, Thermo Fisher Scientific) on a C1000 Touch thermal cycler (Bio-Rad Laboratories). Each 50 µL reaction contained 35.3 µL of nuclease-free water, 5.0 µL of 10× PCR buffer without MgCl_2_, 1.5 µL of 50 mM MgCl_2_ (1.5 mM final), and 1.0 µL of a dNTP mix containing 10 mM of each dNTP (0.2 mM final concentration of each dNTP). The mixture also included 1.0 µL of each primer (10 µM; 0.2 µM final), 0.2 µL of Platinum Taq DNA Polymerase (5 U/µL; 1 U per reaction), and 5.0 µL of template DNA. The thermal cycling programme began with initial denaturation at 94 °C for 2 min, followed by 35 cycles of denaturation at 94 °C for 30 s, annealing at 50 °C for 45 s, and extension at 72 °C for 1 min. Final extension was performed at 72 °C for 7 min, followed by a hold at 12 °C. A sterile-water extraction control and a no-template PCR control were included as described in Section 4.2.

The COI amplicons were sequenced bidirectionally by Sanger sequencing and quality-checked in Geneious R7.1.9 [33]. Fifteen previously published Polish COI sequences from 2020 [19] were included and labelled as previously reported data. All 57 sequences were trimmed to a shared 624-bp homologous region and assigned to the reference haplotypes ES_HP1, ES_HP2, ES_HP4 and EJJ_HP14 using the published mitochondrial lineages [22,23]. Haplotype frequencies were tabulated for the three geographic groups (Table 2), without inferring host population ancestry or migration direction.

### 4.4. Statistical Analyses and Sequence Data Management

COI haplotype frequencies and descriptive haplotype and nucleotide diversity were calculated from the shared 624-bp alignment. The association between host COI haplotype (four categories) and sequence-confirmed infection status (positive or negative) was assessed using the two-sided Fisher–Freeman–Halton exact test for a 4 × 2 contingency table. The exact conditional *p* value was calculated by enumerating tables with fixed margins and summing probabilities no greater than that of the observed table (*p* = 0.712068, reported as *p* = 0.712). The analysis does not establish equivalent infection probabilities among the haplotypes. Wilson 95% confidence intervals describe uncertainty in the observed detection proportions by sampling group. Analyses were implemented in Python 3.14 with Biopython, NumPy, SciPy, pandas and statsmodels [34,35,36,37,38]. These descriptive comparisons were not designed to establish population-genetic equivalence. As the parasite loci were invariant across all 26 infections, no parasite population structure or host–parasite cophylogeny was estimated.

Specimen identifiers were used to link each positive parasite SSU rRNA and ArgRS sequence to its host COI sequence (Supplementary Table S1). Previously published Polish 2020 SSU rRNA and COI records were flagged separately from the new 2022 records and new ArgRS sequences. Public UK records were used for sequence comparison, not treated as a fourth field-sampling group.

Previously published Polish 2020 host COI sequences are available under ON815117–ON815131, and the corresponding *H. eriocheir* SSU rRNA sequences under ON365845–ON365853 [19]. The 2022 host COI accessions are PZ939367–PZ939375 (Poland), PZ939379–PZ939393 (Belgium) and PZ939407–PZ939424 (Germany). The 17 sequence-confirmed 2022 SSU rRNA records comprise PZ939361–PZ939365 (DE2 and PL18, PL20, PL21, PL24), OR229855–OR229859 (Belgium) and OR229861–OR229867 (Germany, excluding DE2). The ArgRS sequences from all 26 confirmed infections are deposited under PZ947004–PZ947029. Individual host–parasite accession links and sequence provenance are given in Supplementary Table S1. Published reference accessions, including the British SSU rRNA and ArgRS records and those explicitly reported in Chinese studies, were checked against GenBank records on 20 October 2026. The lack of verified Chinese-origin entries in the records examined should not be interpreted as evidence that such sequences are absent from GenBank.

## 5. Conclusions

This study provides the first individually linked molecular confirmation and SSU rRNA–ArgRS characterisation of *H. eriocheir* infections in Chinese mitten crabs from the German Elbe and Belgian Scheldt catchments. It also documents new sequence-confirmed infections from Poland in 2022. Parasite sequences were identical at both examined loci across all 26 infections, despite variation in host mitochondrial haplotypes. Repeated detection in the Vistula Lagoon is consistent with parasite transport to the Baltic Sea by migrating *E. sinensis*, but the examined loci cannot establish the geographic source, route or number of introductions.

## Figures and Tables

**Table 1:** Sequence-confirmed *Hepatospora eriocheir* infection in the Chinese mitten crab *Eriocheir sinensis*. Values show the number of small subunit ribosomal RNA (SSU rRNA) sequence-positive crabs/number examined (*n*), the observed detection percentage and Wilson 95% confidence interval (CI). The Polish 2020 cohort was previously reported [19]; all other groups were sampled in autumn 2022.

Sampling Group	Positive/*n*	Detection (%)	Wilson 95% CI (%)
Poland 2020	9/15	60.0	35.7–80.2
Poland 2022	4/9	44.4	18.9–73.3
Germany 2022	8/18	44.4	24.6–66.3
Belgium 2022	5/15	33.3	15.2–58.3
Overall	26/57	45.6	33.4–58.4

**Table 2:** Frequencies of mitochondrial cytochrome c oxidase subunit I (COI) haplotypes and genetic diversity in sampled *Eriocheir sinensis*. *Hd*, haplotype diversity; π, nucleotide diversity; *n*, number of crabs examined. The Polish group includes 15 previously studied individuals collected in 2020 [19] and nine sampled in 2022. EJJ_HP14 is an *Eriocheir japonica*-associated mitochondrial haplotype, not evidence of a distinct nuclear host species.

Population	ES_HP1	ES_HP2	ES_HP4	EJJ_HP14	*n*	*Hd*	π
Poland	11	9	4	0	24	0.649	0.00208
Germany	7	10	1	0	18	0.569	0.00182
Belgium	5	1	0	9	15	0.562	0.02241
All	23	20	5	9	57	0.694	0.01307

**Table 3:** Detection of *Hepatospora eriocheir* infection among *Eriocheir sinensis* mitochondrial cytochrome c oxidase subunit I (COI) haplotypes. Fractions represent the number of crabs with sequence-confirmed small subunit ribosomal RNA (SSU rRNA) amplicons/total number examined in each haplotype. EJJ_HP14 denotes an *E. japonica*-associated mitochondrial lineage; small group sizes limit comparisons of infection probability.

COI Haplotype	Positive/Haplotype Count	Detection (%)
ES_HP1	11/23	47.8
ES_HP2	10/20	50.0
ES_HP4	1/5	20.0
EJJ_HP14	4/9	44.4

## Data Availability

Host COI, parasite SSU rRNA and ArgRS accession numbers are listed by specimen in Supplementary Table S1 and summarised in Section 4.4; previously published Polish 2020 records are identified separately. The specimen-level metadata, nucleotide sequence alignments, reference datasets, analysis scripts, derived results, phylogenetic analysis files and photographic documentation supporting this study are publicly available in Zenodo at https://doi.org/10.5281/zenodo.23540987.

## Supplementary Materials

The following supporting information can be downloaded at: https://www.mdpi.com/article/10.3390/ijms27219646/s1.
